# Pulmonary infections prime the development of subsequent ICU-acquired pneumonia in septic shock

**DOI:** 10.1186/s13613-019-0515-x

**Published:** 2019-03-15

**Authors:** Jean-François Llitjos, Aïcha Gassama, Julien Charpentier, Jérôme Lambert, Charles de Roquetaillade, Alain Cariou, Jean-Daniel Chiche, Jean-Paul Mira, Matthieu Jamme, Frédéric Pène

**Affiliations:** 1 Service de médecine intensive-réanimation, Hôpital Cochin, Hôpitaux Universitaires Paris-Centre, Assistance Publique - Hôpitaux de Paris, 27 Rue du Faubourg Saint-Jacques, 75014 Paris, France; 2 Université Paris Descartes, Paris, France; 3 INSERM U1016, CNRS UMR 8104, Institut Cochin, Paris, France; 4 Service de bio-informatique médicale, Hôpital Saint-Louis, Assistance Publique - Hôpitaux de Paris, Paris, France; 5 Université Paris Diderot, Paris, France; 6 Urgences néphrologiques et transplantation rénale, Hôpital Tenon, Hôpitaux Universitaires Est-Parisien, Assistance Publique - Hôpitaux de Paris, Paris, France; 7 Université Pierre et Marie Curie, Paris, France

**Keywords:** Septic shock, Immunosuppression, Nosocomial infection, Ventilator-acquired pneumonia

## Abstract

**Purpose:**

To investigate the determinants and the prognosis of intensive care unit (ICU)-acquired pneumonia in patients with septic shock.

**Methods:**

This single-center retrospective study was conducted in a medical ICU in a tertiary care center from January 2008 to December 2016. All consecutive patients diagnosed for septic shock within the first 48 h of ICU admission were included. Patients were classified in three groups: no ICU-acquired infections (no ICU-AI), ICU-acquired pneumonia and non-pulmonary ICU-AI. The determinants of ICU-acquired pneumonia and death were investigated by multivariate competitive risk analysis.

**Results:**

A total of 1021 patients were admitted for septic shock, and 797 patients were alive in the ICU after 48 h of management. The incidence of a first episode of ICU-AI was 31%, distributed into pulmonary (17%) and non-pulmonary ICU-AI (14%). Patients with septic shock caused by pneumonia were at increased risk of further pulmonary ICU-AI with a cumulated incidence of 34.4%. A pulmonary source of the initial septic shock was an independent risk factor for subsequent ICU-acquired pneumonia (cause-specific hazard 2.33, 95% confidence interval [1.55–3.52], *p* < 0.001). ICU-AI were not associated with a higher risk of ICU mortality after adjustment in a multivariate-adjusted cause-specific proportional hazard model.

**Conclusion:**

Septic shock of pulmonary origin may represent a risk factor for subsequent ICU-acquired pneumonia without affecting mortality.

**Electronic supplementary material:**

The online version of this article (10.1186/s13613-019-0515-x) contains supplementary material, which is available to authorized users.

## Introduction

Sepsis is an infection-related life-threatening condition characterized by an initial overwhelming pro-inflammatory response followed by a complex immunosuppressive response [1]. Septic shock, its most severe presentation, remains associated with a crude 40% mortality rate, as well as with numerous short-term and long-term complications. Thanks to early recognition of the disorder as well as prompt institution of antimicrobial treatment and advanced life support, most septic shock patients nowadays survive the early phase of resuscitation but then become highly susceptible to intensive care unit (ICU)-acquired infections (ICU-AI) which account for a major cause of death in this setting [2, 3]. Prevention and management of complications in patients recovering from the primary insult now appear as cornerstone therapeutic challenges to improve the overall prognosis of septic shock.

ICU-acquired pneumonia accounts for the majority of ICU-AI in critically ill patients and is associated with increased mortality and other relevant poor outcomes such as prolonged ventilation and prolonged length of stay in the ICU and in the hospital. Classical risk factors for ICU-acquired pneumonia rely on patients’ characteristics including underlying immune and non-immune comorbid conditions, clinical severity and requirements for invasive ventilatory support [4]. Hence, endotracheal intubation and duration of mechanical ventilation are major determinants of so-called ventilator-associated pneumonia. Furthermore, a new paradigm of post-aggressive immunosuppression has emerged in critically ill patients [5]. Accordingly, sepsis-induced immune dysfunctions of most circulating immune cells have been associated with increased susceptibility to secondary infections in previously immunocompetent patients. Such explorations have been conducted in circulating blood cells for obvious accessibility reasons. The impact of multiple sequential insults within target organs on the subsequent risk of infectious complications remains unclear.

We hypothesize that a primary pulmonary infection may contribute to alterations in lung defense and thereby may favor the development of ICU-acquired pneumonia. The aim of this study is to investigate the determinants and the prognosis of ICU-acquired pneumonia in patients with septic shock, with a particular emphasis on linking primary and secondary infectious insults.

## Patients and methods

### Patients and setting

We performed a retrospective single-center study in a 24-bed medical ICU. All patients aged ≥ 18 years old diagnosed with septic shock within the first 48 h of ICU admission were included. Septic shock was defined as a microbiologically proven or clinically suspected infection, associated with acute circulatory failure requiring vasopressors despite adequate fluid filling. Patients who remained in the ICU after 48 h, and therefore at risk of ICU-acquired infections, formed the core of this study. Patients were classified in three groups: no ICU-acquired infections (no ICU-AI), ICU-acquired pneumonia and non-pulmonary ICU-AI. The ethics committee of the French Intensive Care Society approved the study and waived the need for patients’ consents due to its retrospective observational design (ref. CE SRLF, #16–30).

### Intended management

Patients were treated in accordance with the guidelines of the Surviving Sepsis Campaign [6]. Patients were administered intravenous broad-spectrum antibiotics, depending on the presumed site of infection, previous antibiotic treatment and known colonization with antibiotic-resistant bacteria. Antimicrobial treatment was deescalated to narrower spectrum after identification of the responsible pathogen. Source control measures, such as surgery or removal of infected devices, were applied when necessary. They were subjected to a strict sedation protocol based on the Richmond Agitation-Sedation Scale assessment every 3 h and daily stop of sedatives whenever possible, as well as assessment of weaning criteria twice a day in order to reduce the length under mechanical ventilation. End-of-life decisions to withhold or withdraw life support were taken on collectively when maintenance or increase in life-sustaining therapies was considered as futile by all participants and that death would irremediably occur in a short-term manner. Palliative care was then appropriately delivered in the ICU.

### Definitions

Severity at admission was assessed by the Simplified Acute Physiology Score 2 and the Sequential Organ Failure Assessment (SOFA) scores [7, 8]. Patients were considered immunocompromised if one or more of the following conditions were observed: patients with solid tumors with chemotherapy in the last 3 months or a progressive metastatic disease, hematologic malignancies, solid organ transplantation, HIV infection with or without AIDS, treatment with corticosteroids (> 3 months at any dosage or ≥ 1 mg/kg prednisone equivalent per day for > 7 day), or treatment with other immunosuppressive drugs.

The characteristics of both primary and secondary infections included the source and the causative microorganism if microbiologically documented. ICU-acquired infections were defined as any new onset of probable or definite infection that developed after 48 h from ICU admission [2]. Only the first episode of ICU-acquired infection was considered. ICU-acquired pneumonia was diagnosed according to the American Thoracic Society criteria [9]. Briefly, the diagnosis of pneumonia was based on a Clinical Pulmonary Infectious Score > 6. Patients with clinically suspected ventilator-associated pneumonia were usually subjected to a tracheobronchial aspirate with semiquantitative cultures. The diagnosis of ventilator-associated pneumonia and the decision to initiate or not antimicrobial treatment were discussed on daily rounds. A definite diagnosis of catheter-related bloodstream infection required the growth of the same pathogen from both peripheral blood and catheter tip cultures, or from blood cultures sampled from the catheter and from venous puncture with a differential time to positivity > 120 min. The diagnosis of coagulase-negative *Staphylococcus* bloodstream infections required at least two positive blood cultures with the same pathogen. Urinary tract infections, mostly catheter related, were diagnosed upon the association of systemic manifestations of infection and positive urine bacterial culture at ≥ 10^5^ CFU/mL. Invasive fungal infections were diagnosed according to the current guidelines [10]. The diagnostic work-up for ICU-AI did not change during the study period.

### Statistical analysis

Continuous variables were expressed as median (interquartile range) and categorical variables as numbers (percentages) and were compared by the Kruskal–Wallis’ test, the Pearson’s Chi-square test or the Fisher’s exact test as appropriate. ICU mortality was analyzed through a competitive risk framework, with discharge alive as a competing event. The independent predictors of ICU death were investigated in a multivariate Cox cause-specific model, by performing a stepwise backward and forward variable selections based on Akaike information criteria. The model included variables that were significant at *p* value of less than 0.20 in univariate analysis. Proportional hazard assumption was graphically checked, and potential interactions were tested in the final model. Because ICU-AI is a time-dependent covariate, we performed a Cox cause-specific survival analysis, with ICU-AI and the source of infection as predictors, at each landmark time. Landmarking is a common method recommended for the analysis of time-dependent covariates in time-to-event data. We chose each day from day 3 to day 30 for landmark times. Because multiple statistical tests increase the risk of type I error, we applied the Bonferroni correction to adjust *p* values (*p* value threshold = 0.003).

Determinants of ICU-acquired infections were also analyzed through a competing risk framework, with discharge alive and death in ICU as competing events. Since ICU-acquired infections are defined by the occurrence after 48 h, a landmark analysis was performed on the subset of patients still alive in ICU at day 3. Independent determinants of ICU-acquired infections were investigated in a multivariate analysis using a Cox cause-specific proportional hazard model with time-dependent covariates. All analyses were carried out using R 3.1.1 (R foundation for Statistical Computing Vienna, Austria) with the packages “survival,” “cmprsk,” “survival,” “rms” and “stepwise.”

## Results

### Patients

From January 2008 to December 2016, 1021 patients were admitted for septic shock in our ICU. Among them, 147 (14.4%) died early within 48 h and 77 (7.5%) rapidly improved to be promptly discharged from the ICU, leaving 797 patients alive in the ICU after 48 h of management (Additional file 1: Figure S1). Among them, 337 (42%) patients presented with community-acquired infections and 460 (58%) patients had hospital-acquired infections, with a previous in-hospital length of stay of 5 (1–17) days. Most 48-h survivors had underlying comorbid conditions, including 276 (35%) with prior immunosuppression (Table 1). The lung was the most common source of the primary infection in 50% of patients. Adequacy of initial antimicrobial treatment in microbiologically documented infections was 91%. A large majority of patients were mechanically ventilated (*n* = 691, 87%) with a median length of ventilation of 6 (3–12) days; 213 of the 797 48-h survivors (26.7%) died in the ICU. The independent determinants of ICU mortality are displayed in Table 2 and include demographics (age and gender), underlying comorbid conditions (immunosuppression and cirrhosis) and the extent of organ failures (invasive mechanical ventilation and renal replacement therapy).

**Table 1:** Characteristics of patients

Variables	No ICU-AI (*n* = 548)	Pulmonary ICU-AI (*n* = 139)	Non-pulmonary ICU-AI (*n* = 110)	*p*
Age (years)	67 (56–78)	68 (57–78)	69 (57–78)	0.95
Male gender	335 (61)	110 (79)	61 (55)	< 0.001
Comorbid conditions				
Immunosuppression	191 (35)	44 (32)	41 (37)	0.65
Chronic heart failure	130 (24)	25 (18)	22 (20)	0.31
Diabetes mellitus	111 (20)	22 (16)	22 (20)	0.52
COPD	93 (17)	25 (18)	17 (16)	0.87
Chronic renal failure	82 (15)	16 (11)	15 (14)	0.62
Obesity	59 (11)	11 (8)	13 (12)	0.57
Cirrhosis	57 (10)	19 (14)	15 (14)	0.39
Characteristics on ICU admission				
SAPS2, points	64 (49–81)	71 (54–88)	78 (57–89)	< 0.001
SOFA score, points	9 (6–12)	10 (6–13)	9 (6–13)	0.05
Source of infection				< 0.001
Lung	257 (47)	104 (75)	42 (38)	
Digestive	84 (15)	17 (12)	27 (24)	
Urinary	78 (14)	3 (2)	11 (10)	
Skin and soft tissues	41 (7)	3 (2)	5 (4)	
Catheter	33 (6)	3 (2)	8 (7)	
Others	55 (10)	9 (6)	17 (15)	
Microbiological documentation	440 (80)	112 (80)	95 (86)	
Bacteremia	186 (34)	35 (25)	40 (36)	0.09
Microorganisms				
Gram-negative bacteria	278 (50)	57 (41)	57 (52)	0.08
Gram-positive bacteria	151 (28)	49 (35)	33 (30)	
Fungi	10 (2)	6 (4)	4 (4)	
Mycobacteria	1 (0.2)	0 (0)	1 (1)	
Biological findings				
WBC count (per mm^3^)	13.4 (6.8–22.5)	11.9 (4.7–21.4)	13.6 (7.6–21.9)	0.40
Serum protein level (g/L)	59 (52–66)	62 (51–68)	55 (47–65)	0.01
Serum creatinine level (µmol/L)	136 (85–201)	121 (73–204)	138 (80–241)	0.36
Serum bilirubin level (µmol/L)	12 (8–24)	12.5 (8–25)	13 (8–30)	0.72
Prothrombin time (%)	60 (46–73)	64 (45–75)	59 (41–71)	0.47
ICU management				
Invasive mechanical ventilation	448 (82)	137 (99)	106 (96)	< 0.001
Renal replacement therapy	197 (36)	83 (60)	76 (69)	< 0.001
Surgical source control	136 (25)	18 (13)	28 (25)	0.01
Aminoglycosides	321 (59)	64 (46)	69 (63)	0.01
Stress-dose steroids	228 (42)	70 (53)	64 (59)	< 0.001
Outcomes				
Duration of mechanical ventilation (days)	4.5 (2–8)	18 (10–29.5)	13 (8–21)	< 0.001
End-of-life decision	66 (12)	33 (24)	29 (26)	< 0.001
ICU mortality	99 (18)	64 (46)	50 (45)	< 0.001

Variables are expressed as median (interquartile range) or number (percentage) as appropriate

*ICU* intensive care unit, *ICU-AI* intensive care unit-acquired infections, *COPD* chronic obstructive pulmonary disease, *SAPS2* Simplified Acute Physiology Score 2, *SOFA* Sequential Organ Failure Assessment, *WBC* white blood cell

**Table 2:** Determinants associated with ICU mortality using multivariate Cox cause-specific model

	CSH ratio	95% CI	*p*
Age			
0–59 years	Ref.	Ref.	Ref.
60–74 years	1.48	1.02–2.14	0.04
> 75 years	2.20	1.54–3.16	< 0.001
Male gender	0.75	0.56–0.99	0.05
Immunocompromised status	1.63	1.23–2.16	< 0.001
Cirrhosis	1.55	1.05–2.26	0.02
Mechanical ventilation	3.14	0.99–9.94	0.05
Renal replacement therapy	1.91	1.39–2.65	< 0.001

*CSH ratio* cause-specific hazard ratio; *95% CI* 95% confidence interval

### Characteristics and outcome of ICU-acquired infections

**Fig. 1:**
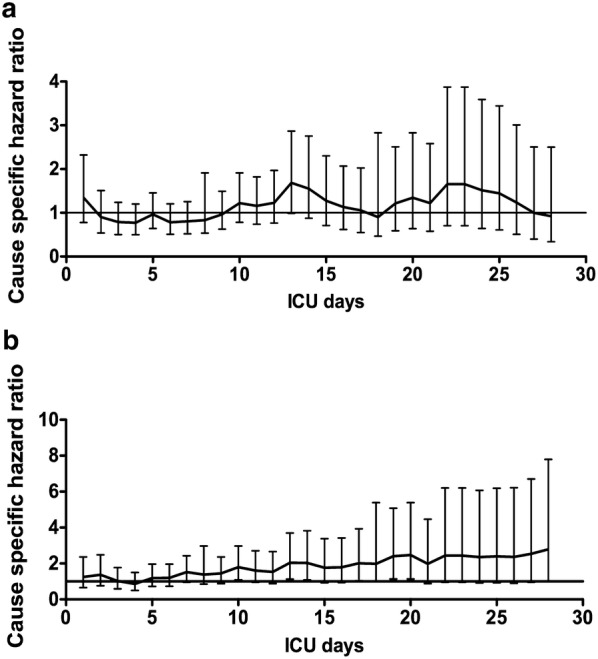
Effects of pulmonary (**a**) and non-pulmonary (**b**) ICU-acquired infections on ICU mortality. The figure displays the risk of death (cause-specific hazard ratio with 95% confidence interval) associated with ICU-acquired infections in patients still at risk (alive in the ICU) at each time points (landmarks)

Among 48-h survivors, the incidence of a first episode of ICU-AI was 31% (*n* = 249), distributed into pulmonary (*n* = 139, 17%) and non-pulmonary ICU-AI (*n* = 110, 14%). Characteristics of ICU-AI are displayed in Table 3. Microbiological documentations of primary and ICU-acquired pneumonia were all different in terms of strains and/or antibiotic susceptibility. Non-pulmonary ICU-AI episodes were mainly related to catheter-related infections (43%) and abdominal infections (31%). Time from admission to ICU-AI diagnosis was similar for both pulmonary and non-pulmonary ICU-AI, 7 (5–11) days and 8 (5–12) days, respectively. Patients with ICU-AI exhibited higher SAPS2 severity score at ICU admission resulting in higher requirements for stress-dose steroids, invasive mechanical ventilation and renal replacement therapy (Table 1). Of note, the underlying immune status did not impact on the susceptibility to ICU-AI. The occurrence of ICU-AI was associated with protracted invasive mechanical ventilation, especially if pulmonary ICU-AI, and a marked increase in ICU mortality (46% vs. 18% in patients without ICU-AI, *p* < 0.001). However, neither pulmonary nor non-pulmonary ICU-AI remained significantly associated with a higher risk of ICU mortality in a multivariate-adjusted cause-specific proportional hazard model (Fig. 1).

**Table 3:** Characteristics of ICU-acquired infections

Variables	Pulmonary ICU-AI (*n* = 139)	Non-pulmonary ICU-AI (*n* = 110)	*p*
Time from admission to ICU-AI (days)	7 (5–11)	8 (5–12)	0.57
Sources of non-pulmonary ICU-AI			
Catheter	–	47 (43)	
Abdominal	–	34 (31)	
Skin and soft tissue	–	15 (14)	
Urinary	–	5 (4)	
Miscellaneous	–	3 (3)	
Unknown	–	6 (5)	
Microbiological documentation	112 (80)	85 (77)	0.10
Positive blood culture	11 (8)	61 (55)	< 0.001
Distribution of pathogens			< 0.001
Gram-negative bacteria			
*Pseudomonas aeruginosa*	39 (28)	10 (9)	
*Escherichia coli*	18 (13)	3 (2)	
*Enterobacter* spp.	15 (11)	13 (12)	
*Klebsiella* spp.	13 (9)	7 (6)	
*Serratia marcescens*	7 (5)	0 (0)	
Miscellaneous	7 (5)	1 (1)	
Gram-positive bacteria			
*Staphylococcus aureus*	6 (4)	8 (7)	
*Enterococcus* spp.	1 (1)	11 (10)	
Miscellaneous	2 (2)	20 (19)	
Fungi			
*Candida albicans*	0 (0)	3 (2)	
*Non*-*albicans Candida*	0 (0)	6 (5)	
*Aspergillus fumigatus*	2 (1)	0 (0)	
Miscellaneous	2 (2)	0 (0)	
Virus			
*Cytomegalovirus*	0 (0)	3 (2)	
Further episodes of ICU-AI			0.08
None	85 (61)	72 (65)	
1 episode	25 (18)	26 (24)	
≥ 2 episodes	29 (21)	12 (11)	

Variables are expressed as median (interquartile range) or number (percentage), as appropriate

*ICU* intensive care unit, *ICU-AI* intensive care unit-acquired infections, *MRSA* methicillin-resistant *Staphylococcus aureus*, *MSSA* methicillin-susceptible *Staphylococcus aureus*, *GNB* gram-negative bacteria

### Determinants of pulmonary ICU-AI

**Fig. 2:**
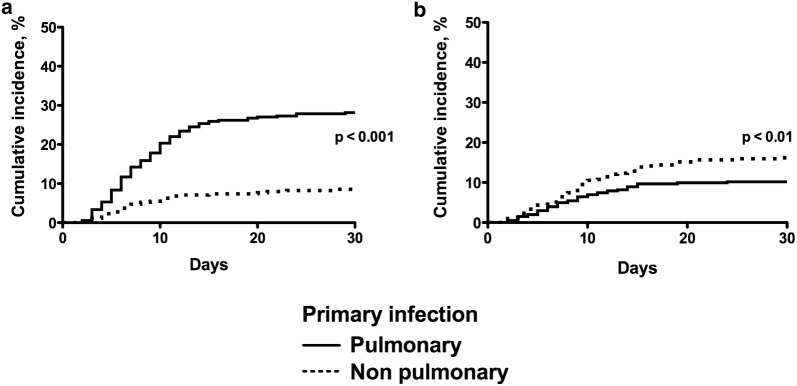
Cumulative incidence of pulmonary (**a**) and non-pulmonary (**b**) ICU-acquired infections depending on the source of primary infection

Most importantly, we observed a link between sources of primary and secondary infections. Thus, patients with septic shock caused by pneumonia carried a particular susceptibility to further pulmonary ICU-AI with a cumulated incidence of 34.4% (Fig. 2a). In contrast, a primary pneumonia did not favor the occurrence of non-pulmonary ICU-AI (Fig. 2b). Differences between patients with ICU-acquired pneumonia and other patients without ICU-AI or with non-pulmonary ICU-AI are displayed in Table 1.

Using a cause-specific Cox regression multivariate model, we plotted the pulmonary source of the primary infection against other relevant determinants of ICU-acquired pneumonia including prior invasive mechanical ventilation treated as a time-dependent covariate (Table 4). A primary pulmonary infection was an independent risk factor for subsequent ICU-acquired pneumonia (pulmonary vs. non-pulmonary primary infections: CSH 2.33, 95% confidence interval [1.55–3.52], *p* < 0.001). Similar results were obtained in a sensitivity analysis performed in the 691 patients who had required invasive mechanical ventilation (pulmonary vs. non-pulmonary primary infections: CSH 2.03, 95% confidence interval [1.35–3.05], *p* < 0.001).

**Table 4:** Determinants associated with ICU-acquired pneumonia

Variables	Univariate			Multivariate		
	CSH ratio	95% CI	*p*	CSH ratio	95% CI	*p*
Male gender	2.14	1.41–3.25	< 0.001	2.17	1.35–3.45	0.001
Source of primary infection						
Lung	Ref.	Ref.	Ref.	Ref.	Ref.	Ref.
Digestive	0.49	0.28–0.86	0.01	0.59	0.33–1.05	0.07
Urinary	0.24	0.07–0.76	0.01	0.29	0.09–0.89	0.03
Skin and soft tissue	0.29	0.09–0.93	0.03	0.33	0.10–1.04	0.06
Catheter	0.36	0.11–1.14	0.08	0.47	0.15–1.48	0.19
Others	0.43	0.20–0.88	0.02	0.49	0.23–1.02	0.05
Creatininemia	0.99	0.99–1	0.10			
SOFA score	1.03	1–1.06	0.04	1.03	1.01–1.07	0.03
Mechanical ventilation	1.16	0.47–2.84	0.75			
Aminoglycosides	0.67	0.47–0.94	0.02			

All covariates were analyzed at baseline status except for mechanical ventilation which was evaluated as time-dependent covariate

*CSH ratio* cause-specific hazard ratio, *95% CI* 95% confidence interval, *SOFA* Sequential Organ Failure Assessment

## Discussion

We herein reported that ICU-acquired pneumonia account for the majority of secondary infections in patients who survived the early days of septic shock. After adjustment with potential confounders, including the exposition to invasive mechanical ventilation, a primary pulmonary infection was independently associated with the development of further ICU-acquired pneumonia. This suggests that a primary pulmonary insult may impair lung defense mechanisms toward secondary infections.

Recent cohorts of sepsis and septic shock patients have reported that ICU-acquired infections remain frequent and dreaded complications, with incidence ranging from 13.5 to 23% [2, 11]. Incidence of nosocomial infections is higher in critical care setting than in general hospital wards given the complex interactions between underlying diseases, the severity of acute illness and the multiplicity and the maintenance of invasive devices [12, 13]. We reported here a slightly higher incidence ICU-AI of 31%, with a majority of nosocomial pneumonia, probably linked to the underlying characteristics of our cohort. Although ICU-AI are clearly associated with a higher risk of death in critically ill patients, whether they directly contribute to mortality or only account for a surrogate marker of frailty remains questionable. Moreover, the burden of antimicrobial resistance is increasing, due to extensive antimicrobial use and protracted hospital length of stay, and raises new therapeutic challenges. Hence, early identification of ICU-AI and infection control measures are of major concern in preventing ICU-AI and decreasing the spread of antimicrobial resistance.

Although the development of ICU-AI is commonly associated with an increased risk of death, multiple additional factors are likely to contribute to mortality in critically ill patients including underlying comorbid conditions, accumulation of organ failures, infectious and non-infectious complications. This may frequently result in therapeutic limitations that may prevent appropriate diagnostic work-up and treatment. Sophisticated statistical models taking into account such confounders allow addressing the specific attributable mortality of nosocomial infections, defined as the percentage of death that would not have occurred without infection. Thus, recent studies retrieved little impact of ICU-acquired infections, and more specifically of ventilator-associated pneumonia, on ICU mortality [2, 14, 15]. Using a multivariate cause-specific hazard model, we observed that both pulmonary ICU-AI and non-pulmonary ICU-AI were not significantly associated with a higher risk of ICU mortality.

The major result of this study lies in the link between primary and secondary sources of infection. Specifically, we found that the pulmonary source of the primary infection was a risk factor for further ICU-acquired pneumonia, independent from prior requirement and duration of invasive mechanical ventilation. This result suggests regional disparities induced by different sources of infection that rely on the compartmentalization of immune responses. Primary pulmonary or non-pulmonary insults may differentially impact on lung defense and modulate the further susceptibility to superimposed infectious insults. The regimen and duration of prior antibiotics may also impact on the type of pathogens involved in ventilator-associated pneumonia and their eventual susceptibility to antimicrobials [16]. We also found that male patients had an increased risk of developing pulmonary ICU-AI. We could hardly find a definite explanation, but it is likely that sexual hormones play a role in the regulation of the immune system and may therefore impact on the eventual susceptibility to and outcome of sepsis [17].

It is now well documented that both pulmonary and non-pulmonary sepsis may induce various quantitative and functional changes in most immune cells [18], which have been associated with an increased susceptibility to secondary infections [19–22]. Furthermore, experimental studies have highlighted that different body compartments may not behave similarly following an infectious insult. For instance, alveolar macrophages do not produce interferon-β after LPS stimulation when compared to peritoneal macrophages [23]. Therefore, assuming a regional compartmentalization of immune response, several mechanisms could participate to explain the susceptibility of the lung to a second infectious hit after septic shock of pulmonary origin. A recent study reported acquired tolerogenic properties of pulmonary dendritic cells, an important subset of antigen-presenting cells, following severe primary pulmonary infection with an increased susceptibility to secondary infections [24]. Besides sepsis-induced immunosuppression, various ICU interventions such as mechanical ventilation or systemic therapeutics such as antimicrobials, corticosteroids and transfusions may contribute to alter lung susceptibility to secondary infections. Since mechanical ventilation is a well-known risk factor for ventilator-associated pneumonia [9], pulmonary alterations due to mechanical ventilation may participate to lung susceptibility to secondary infections. In experimental models, ventilated injured lungs develop direct damage in both epithelial and endothelial cells [25]. Another interesting line of approach is the relation between microbiota and antimicrobial treatment. A recent experimental study provides data suggesting that both intestinal and upper airway microbiota promote the innate response to pulmonary infections by enhancing pathogens clearance [26]. One could hypothesize that a previous episode of pneumonia could modify the bacterial composition of respiratory microbiota and therefore promote susceptibility to secondary infections.

As already reported in a previous study involving part of the present cohort, we observed that the underlying immune status did not confer a higher susceptibility toward ICU-acquired infections [27]. Although this observation may contradict a common belief, several explanations can be proposed. All septic shock patients were exposed to invasive procedures and breakthrough of natural barriers to infections. Most non-immunocompromised patients exhibited non-immune comorbidities also likely to impair local and/or systemic anti-infectious defense mechanisms. Finally, sepsis-induced dysfunctions can promote profound immunosuppression smoothing away the immune capacities between immunocompromised and non-immunocompromised patients.

This study has several limitations. First, this was a retrospective study, performed in a single center with a particular case mix of patients and a high prevalence of immune comorbidities. Such a study can only identify association but not causality links between variables. Second, we were able to collect only ICU-acquired infections, but we did not address the incidence of further hospital-acquired infections following ICU discharge. Third, the study did not include a control group of patients with primary pneumonia but no septic shock, which may allow understanding the respective roles of pulmonary insults and shock on the further susceptibility to pulmonary ICU-AI. In addition, some potential confounders such as prior pulmonary lesions and prior antimicrobial exposure may also impact on the risk of pulmonary ICU-AI. Fourth, we focused on the first episode of ICU-acquired infection, whereas one third of those patients exhibited additional infection episodes throughout the ICU stay.

## Conclusion

A prior episode of pulmonary infection in patients with septic shock may represent a major risk factor for secondary ICU-acquired pneumonia. A better understanding of pathophysiological mechanisms of post-septic alterations in lung defense could contribute to better stratify the risk of secondary pneumonia, and therefore identify patients likely to benefit from innovative preventive or therapeutic approaches.

## Additional file

**Additional file 1: Figure S1.** Flowchart of the study. ICU: intensive care unit.

## Abbreviations

HVEM: herpes virus entry mediator
ICU-AI: ICU-acquired infections
LPS: lipopolysaccharide
SOFA: Sequential Organ Failure Assessment
