# Supplementary figures and images for: Pulmonary infections prime the development of subsequent ICU-acquired pneumonia in septic shock
Source: Ann Intensive Care. 2019 Mar 15;9:39. doi: 10.1186/s13613-019-0515-x (PMC6420540; doi:10.1186/s13613-019-0515-x)

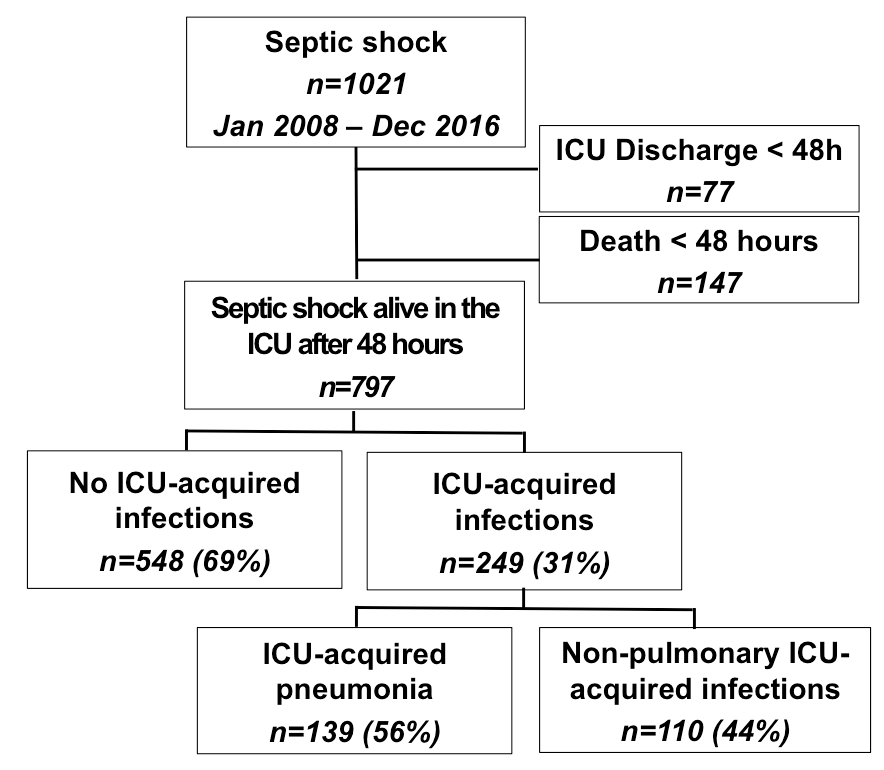


**Figure S1:** Flow-chart of the study. ICU: Intensive care unit.

Supplement: Supplementary file 1 — Additional file 1: Figure S1. Flowchart of the study. ICU: intensive care unit. [file 13613_2019_515_MOESM1_ESM.docx]
